# Downregulation of GAS5 Promotes Bladder Cancer Cell Proliferation, Partly by Regulating CDK6

**DOI:** 10.1371/journal.pone.0073991

**Published:** 2013-09-17

**Authors:** Zhihong Liu, Wei Wang, Juntao Jiang, Erdun Bao, Dongliang Xu, Yigang Zeng, Le Tao, Jianxin Qiu

**Affiliations:** Department of Urology, Shanghai First People’s Hospital, Medical College of Shanghai Jiao Tong University, Shanghai, China; Biomedical Research Foundation, Academy of Athens, Greece

## Abstract

Long non-coding RNAs (lncRNAs) play important roles in diverse biological processes, such as transcriptional regulation, cell growth and tumorigenesis. However, little is known about whether lncRNA-GAS5 (growth arrest-specific 5) regulates bladder cancer progression. In the present study, we found that the GAS5 expression is commonly downregulated in bladder cancer cell lines and human specimens. Knockdown of GAS5 promotes bladder cancer cell proliferation, whereas forced expression of GAS5 suppresses cell proliferation. We further demonstrated that knockdown of GAS5 increases CDK6 mRNA and protein levels in bladder cancer cells. Expectedly, GAS5 inhibition induces a significant decrease in G0/G1 phase and an obvious increase in S phase. Gain-of-function and loss-of-function studies showed that GAS5 inhibits bladder cancer cell proliferation, at least in part, by regulating CDK6 expression.

**Conclusions:**

Downregulated GAS5 promotes bladder cancer cell proliferation, partly by regulating CDK6, and thus may be helpful in the development of effective treatment strategies against bladder cancer.

## Introduction

Human bladder cancer is the fourth most common malignancy in men, and the tenth most common in women [1], [2]. The most common histological type of bladder cancer is urothelial carcinoma (UC) which are non-invasive papillary tumors that commonly recur but rarely progress [3]. In general, the treatment for these patients is endoscopic resection [4], [5]. Invasive bladder tumors are more aggressive, and patients with muscle invasive UC are usually treated with radical cystectomy. However one-half of patients with invasive bladder cancer develop subsequent metastatic disease, even after radical surgery of the primary tumors [6]. The advances in effective therapy for bladder cancer have been limited because the pathological mechanisms causing tumor are not known. Therefore, revealing the molecular mechanism for the bladder tumorigenesis is indispensable for developing effective treatment.

Recent evidence shows that long non-coding RNAs (lncRNAs) play important roles in diverse biological processes, such as transcriptional regulation, cell growth and tumorigenesis [7], [8]. Our previous studies showed that H19 increases bladder cancer cell proliferation and metastasis [9], [10]. Upregulated H19 contributes to bladder cancer growth by regulating ID2 expression. Upregulated H19 also increases bladder cancer cell metastasis by associating EZH2 and inhibiting E-cad expression. The HOTAIR (for HOX antisense intergenic RNA) level is increased in primary tumors and regulates cancer progression [11]. Overexpression of HOTAIR in epithelial cancer cells leads to altered histone H3 lysine 27 methylation and promotes cancer metastasis [12]. HOTAIR is a critical element in metastatic progression by associating with members of the PRC2 complex (SUZ12, EZH2, and H3K27me3) [13], [14]. LncRNA-MEG3 (maternally expressed gene 3) is also associated with tumorigenesis and progression of meningioma [7]. MEG3 is not expressed in the majority of human meningiomas or the human meningioma cell lines [7]. Re-expression of MEG3 inhibits tumor cell proliferation and colony formation in soft agar by inducing accumulation of p53 protein and selectively regulating p53 target gene expression [15]. GAS5 is a recently identified non-coding RNA which is associated with cell proliferation. GAS5 plays an essential role in normal growth arrest in both T-cell lines and non-transformed lymphocytes [16]. Overexpression of GAS5 reduces the rate of progression through the cell-cycle, whereas downregulation of GAS5 inhibits apoptosis and maintains a more rapid cell cycle. Mourtada-M *et al* demonstrated that GAS5 transcript levels are significantly decreased in breast cancer samples compared to adjacent normal breast epithelial tissues [17]. Overexpression of GAS5 induces growth arrest and apoptosis independently of other stimuli. However, the underlying mechanisms for GAS5 regulating cancer cell proliferation remain unclear.

Based on these findings, we tested whether GAS5 regulates cell cycle and cell proliferation in bladder cancer cell. We found that GAS5 expression is significantly decreased in bladder cancer tissues. Knockdown of GAS5 induces a significant decrease in G0/G1 phase and promotes bladder cancer cell proliferation, at least in part, by regulating CDK6 expression.

## Materials and Methods

### Tissue Samples and Cell Lines

Human bladder tissues were obtained with written informed consent from the First People’s Hospital affiliated to School of Medicine Shanghai Jiaotong University. The study was approved by the Ethics Committee of the School of Medicine Shanghai Jiaotong University. 28 specimens of bladder cancer tissue and their adjacent normal tissues were collected between 02/2011 and 12/2012 (Table 1).

**Table 1:** The characteristics of patients with bladder cancer.

Gender	28
Male (%)	19 (68%)
Female (%)	9 (32%)
Mean age	65 (45–78)
T stage	
Ta	6
T1	6
T2	9
T3	5
T4	2
N stage	
N0N1	17
higher	11
Grade	
Grade 1–2	6
Grade3	14
Grade4	8

Human bladder cancer cells (T24, DSH1, RT112, RT4, KU7 and 253J cells) were obtained from the American Type Culture Collection (ATCC, Manassas, VA) and cultured in RPMI 1640 (Gibco, Carlsbad, CA,) with 10% fetal bovine serum (FBS; Gibco).

### Real-time Polymerase Chain Reaction (PCR)

Total RNA was extracted from bladder cancer tissues or cells by using Trizol reagent (Invitrogen, Carlsbad, CA), and the reverse transcription reactions were performed using random primers and an M-MLV Reverse Transcriptase kit (Invitrogen). Real-time PCR was carried out using a standard protocol from the SYBR Green PCR kit (Toyobo, Osaka, Japan) on Applied Biosystems 7300 Real Time PCR system (Applied Biosystems, Foster City, CA) according to the instructions. β-actin was used as references for lncRNAs. ΔCt values were normalized to β-actin levels. Each sample was analyzed in triplicate.

### Western Blot Analysis

Western blot analysis to assess CDK6 and β-actin expression was carried out as previously described [18]. The anti-CDK6 primary antibodies were purchased from Santa Cruz Biotechnology (CA, USA). β-actin primary antibodies were purchased from Sigma (MO, USA).

### Flow Cytometric Analysis

RT4 cells (1∼2×10^5^) treated with GAS5-siRNA or CDK6-siRNA were plated in 6-well plates. After 48-hour incubation, the cultures were incubated with propidium iodide for 30 min in the dark. Cultures were collected and analyzed for cell cycle using a flow cytometer (FACSCalibur, BD Biosciences) after propidium iodide staining. The cultures were also stained with annexin V–fluorescein isothiocyanate, and the cell apoptosis was analyzed using a flow cytometer.

### Overexpression and Small Interfering RNA

To express GAS5, plasmid pcDNA-GAS5 was constructed by introducing a *KpnI-XhoI* fragment containing the GAS5 cDNA into the same sites in pcDNA3.1. The GAS5 gene was amplified from cDNA prepared from T24 cells by PCR using the forward and reverse primers: ggggtaccTTTCGAGGTAGGAGTCGAC and ccgctcgagGGATTGCAAAAATTTATTAAAATTG. pcDNA-GAS5 was transfected into bladder cancer cell line by using Lipofectamine 2000 (Invitrogen).

To inhibit endogenous GAS5 and CDK6 expression, 2×10^5^ cells per well in a six-well plate were transfected with 50 nM indicated siRNA or negative control using Lipofectamine 2000. Then cells were incubated with siRNA for the indicated time. Three different GAS5-siRNAs (reference sequence NR_002578) were designed by Ambion (Ambion, Austin, TX). The CDK6-siRNAs used in this study were mixtures of three siRNAs and were purchased from Ambion.

### Cell Proliferation Assay

Cell proliferation assays were carried out using Cell Counting Kit-8 kit (Dojindo Laboratories, Kumamoto, Japan). RT4 cells were plated in 24-well plates in triplicate at approximately 1×10^5^ cells per well and cultured in the growth medium. RT4 cells were then treated with pcDNA-GAS5 or GAS5-siRNA, and the numbers of cells per well were measured by the absorbance (450 nm) of reduced WST-8 (2-(2-methoxy-4-nitrophenyl)-3-(4-nitrophenyl)-5-(2,4-isulfophenyl)-2H-tetrazolium, monosodium salt) at the indicated time points.

### RNA Immunoprecipitation and RNA Pulldown

RNA immunoprecipitation (RIP) or RNA Pulldown was performed as described previously [10]. Briefly, for RNA pulldown assay, biotin-labeled RNAs were *in vitro* transcribed with the Biotin RNA Labeling Mix (Roche Diagnostic, Indianapolis, USA) and T7 RNA polymerase (Roche). Cell nuclear extract (2 µg) was mixed with biotinylated RNA (100 pmol). Washed Streptavidin agarose beads (100 µl) were added to each binding reaction and further incubated at room temperature for 1 h. Beads were washed briefly three times and boiled in SDS buffer, and the retrieved protein was detected by standard western blot technique.

The CDK6 antibodies used for RIP are purchased from Abcam (Abcam, Cambridge, MA). The coprecipitated RNAs were detected by reverse transcription PCR. Total RNAs and controls were also assayed to demonstrate that the detected signals were from RNAs specifically binding to CDK6.

### Statistical Analysis

Statistical comparison between 2 groups was performed using unpaired *t*-test. All of the groups were compared using one-way analysis of variance (ANOVA), followed by Tukey post hoc test where appropriate. The difference was deemed statistically significant at *p*<0.05. All data were represented as mean ± standard deviateon from at least three separate experiments.

## Results

### GAS5 Level is Significantly Downregulated in Bladder Cancer

**Figure 1:**
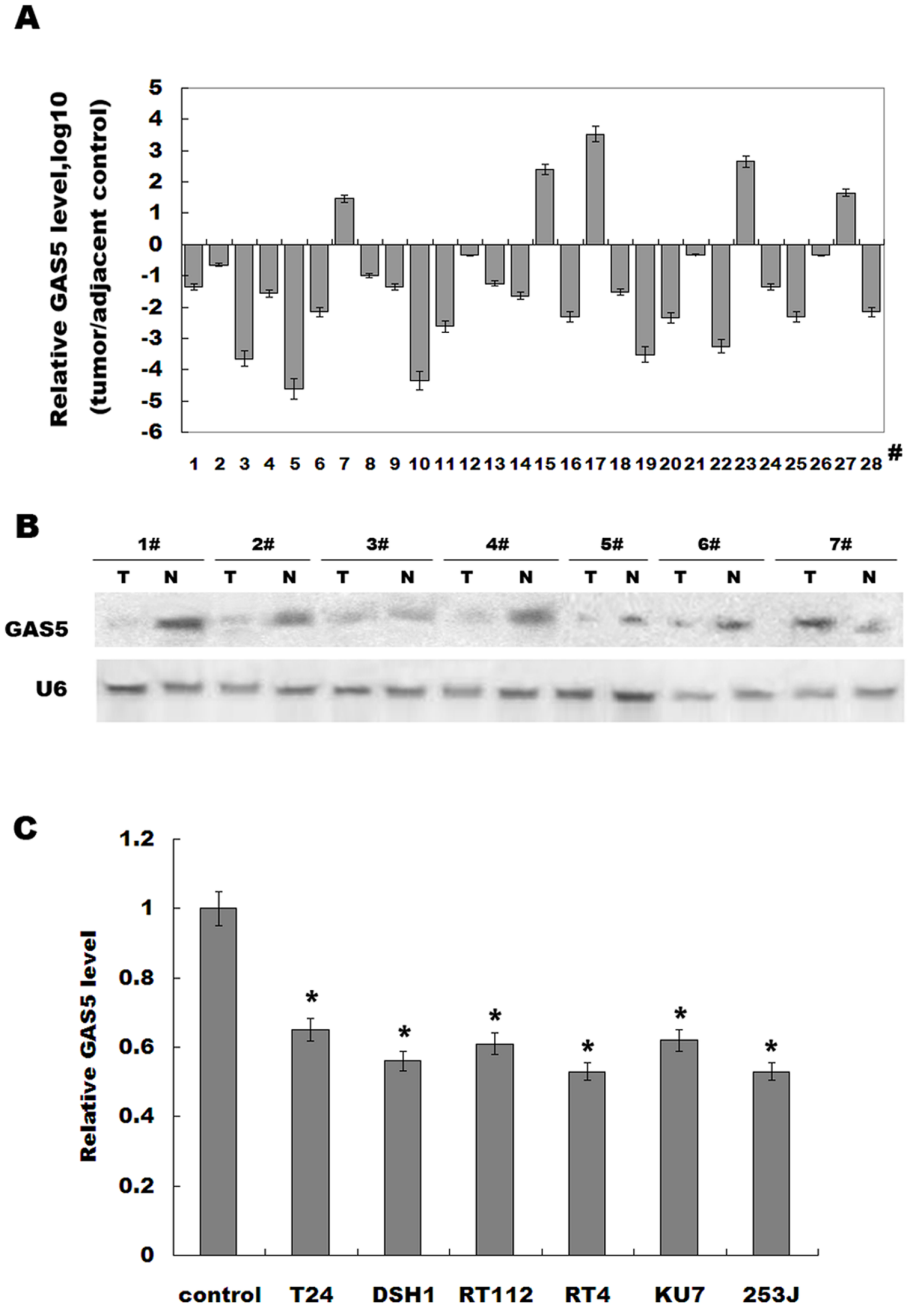
GAS5 levels are decreased in bladder cancer. (A) Analysis of GAS5 expression level was carried out in bladder cancer tissues or adjacent control (n = 28). Total RNA was subjected to real-time RT-PCR to analyze the CT values of bladder cancer normalized to β-actin in each sample. The normalized values (ΔCT) from all tissues were then compared with a normal tissue, in each group (ΔΔCT). The results were expressed as Log10 (2^–ΔΔCt^). (B) RNA of 1–7# used in (A) was assayed by northern blot analysis as previously described [32]. (C) GAS5 levels were evaluated by real-time PCR in bladder cancer cell lines. Normal urothelial cells were used as control. *p<*0.05*.

Previous studies showed that GAS5 controls cell apoptosis and is downregulated in breast cancer [17]. In order to investigate whether GAS5 regulates bladder tumorigenesis, we first examined the GAS5 expression level in bladder cancer tissues and adjacent normal tissues. Figure 1A showed that GAS5 expression is remarkably downregulated in 82% bladder cancer tissues compared with adjacent controls. To confirm the validity of GAS5 reduction, a portion of the RNA used for the real-time PCR was subjected to northern blotting analysis. Consistent with the above findings, GAS5 was reduced in most bladder cancer tissues (Figure 1B). We then examined the expression level of GAS5 in bladder cancer cell lines (T24, DSH1, RT112, RT4, 253J, and KU7). Compared to the normal urothelia cell, GAS5 expression is significantly decreased in these bladder cancer cell lines (Figure 1C). These data indicate that downregulation of GAS5 may be related to bladder cancer progression.

### GAS5 Inhibits Bladder Cell Proliferation *in vitro*

**Figure 2:**
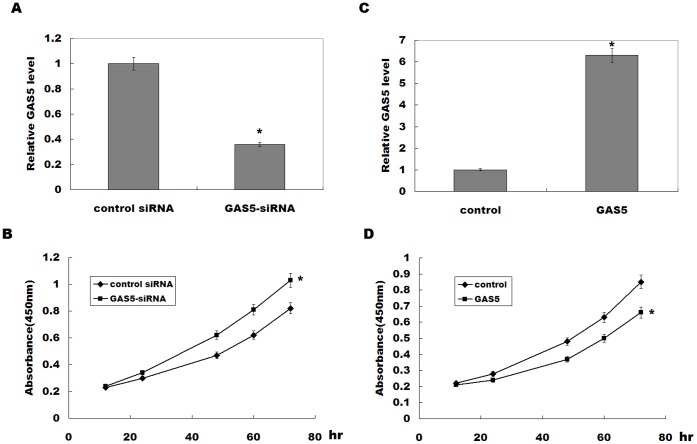
GAS5 inhibits bladder tumor cell proliferation. (A) RT4 cells were treated with GAS5-siRNA, and GAS5 expression level was assayed after 48 h by real-time PCR. (B) RT4 cells were treated with GAS5-siRNA, and at the indicated time points, the numbers of cells per well were measured by the absorbance (450 nm) of reduced WST-8. (C) RT4 cells were treated with pcDNA-GAS5, and GAS5 expression level was assayed by real-time PCR. (D) RT4 cells were transiently overexpressed with GAS5, and the numbers of cells per well were measured by the absorbance (450 nm) of reduced WST-8. The results show data from at least three independent experiments, expressed as the mean ± SD. *P<*0.05*.

To investigate the biological role of GAS5 in regulating bladder cancer cell proliferation, the bladder cancer cell treated with GAS5 or GAS5-siRNA were analyzed. GAS5-siRNA treatment significantly inhibits GAS5 expression, and GAS5 knockdown increases RT4 cell proliferation (Figure 2A and B). Oppositely, pcDNA-GAS5 treatment upregulates GAS5 expression, and suppresses RT4 cell proliferation (Figure 2C and D). These data suggest that downregulated GAS5 in bladder cancer contributes to bladder cancer cell proliferation.

### GAS5 Negatively Regulates CDK6 Expression *in vitro*

**Figure 3:**
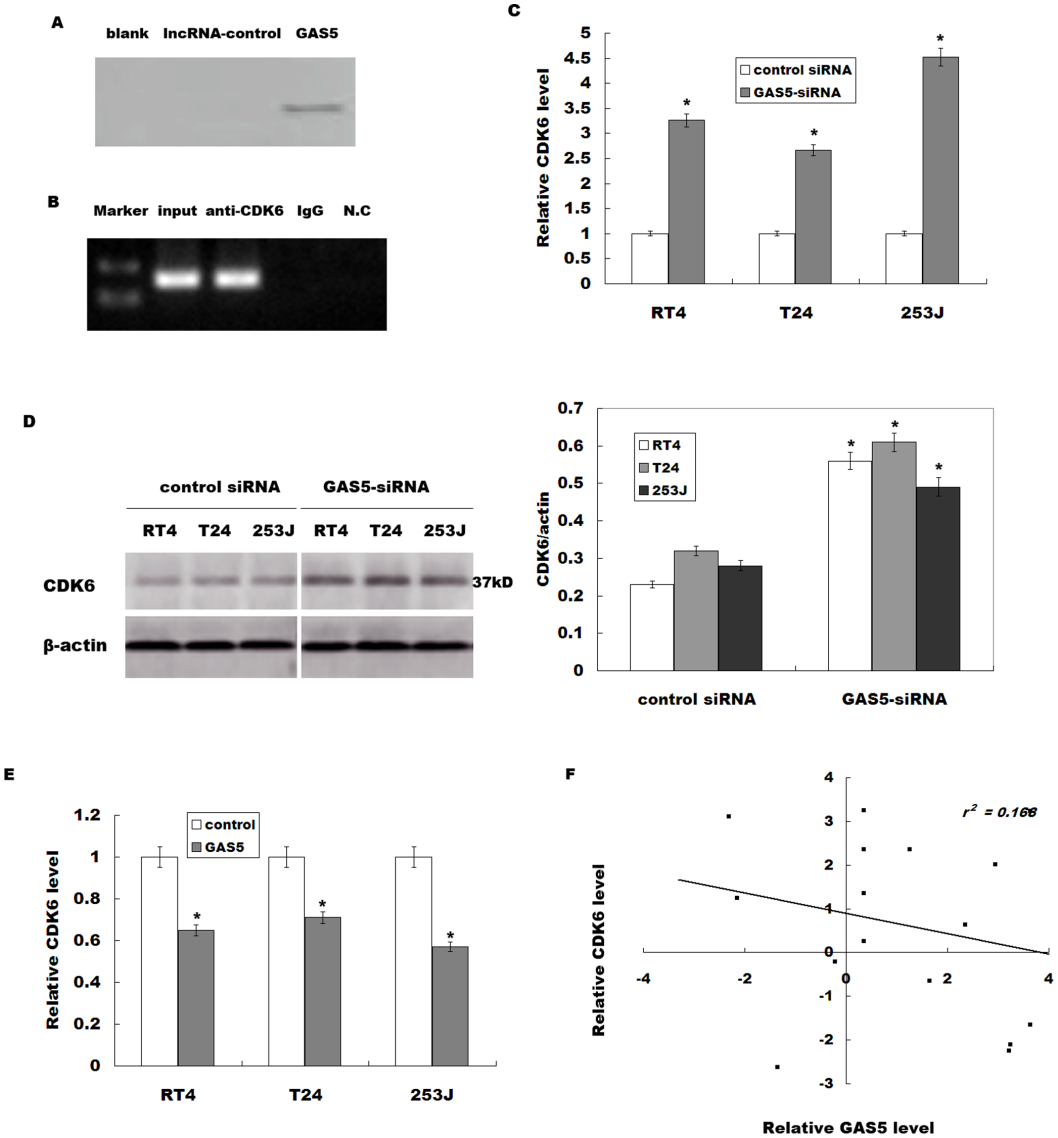
GAS5 negatively regulates CDK6 expression. (A) Biotinylated GAS5 or control were incubated with nuclear extracts (RT4 cells), targeted with streptavidin beads and associated proteins were resolved in a gel. Silver staining of the SDS-PAGE gel containing aliquots of samples derived from proteins pulled down by GAS5. (B) RIP experiments were performed using CDK6 antibody to immunoprecipitate RNA and a primer to detect GAS5 RNA. (C) Analysis of CDK6 mRNA level was performed in bladder cancer cell lines after GAS5-siRNA treatment. (D) Western blot analysis of CDK6 protein level was performed in bladder cancer cells treated with GAS5-siRNA. (E) Analysis of CDK6 mRNA level was performed in bladder cancer cell lines after GAS5 overexpression. The results show data from at least three independent experiments, expressed as the mean ± SD. *P<*0.05*. (F) Negative correlation between GAS5 levels and the CDK6 levels in 16 bladder cancer samples (*r* ^2^ = 0.168, *p = 0.013*).

**Figure 4:**
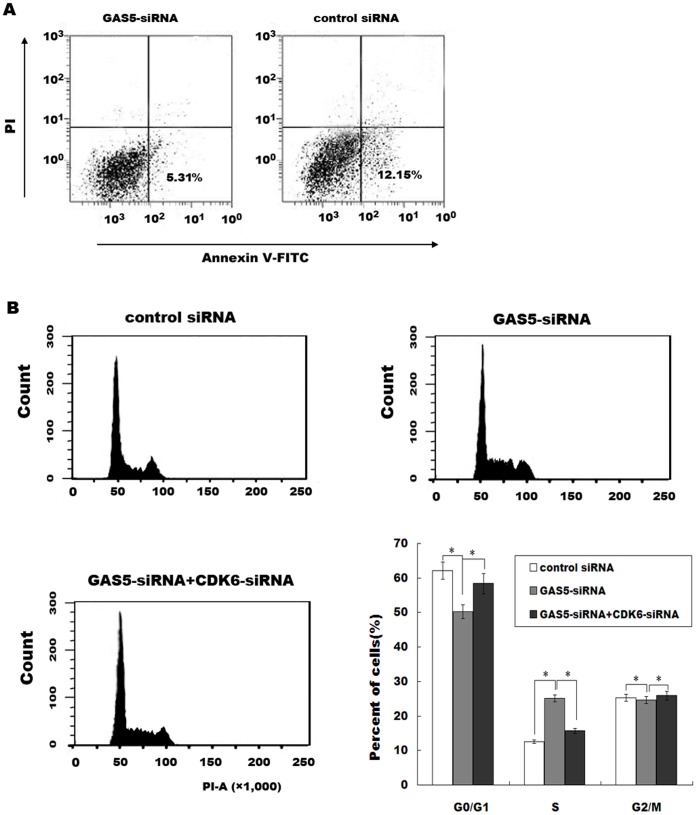
GAS5 regulates bladder cancer cell cycle by regulating CDK6. (A) GAS5 expression was inhibited by specific siRNAs in RT4, and the cell apoptosis was analyzed by flow cytometer 48 h later. (B) RT4 cells were treated with GAS5-siRNA or CDK6-siRNA. Forty-eight hours later, the relative cell numbers in each cell cycle phase after propidium iodide staining were determined by FACS analysis. The data are from one of three independent experiments. The histograms were analyzed and the percentage of cells in each phase of the cell cycle is shown. The results are presented as mean ± SD for three experiments.

We then investigated the possible mechanisms that GAS5 regulates the bladder cancer cell proliferation. We performed an RNA pull-down assay to identify proteins that associated with GAS5 (Figure 3A). Mass spectrometry analysis of the protein band specific to GAS5 revealed that Cyclin-dependent kinase 6 (CDK6) is specifically associated with GAS5 (Table 2). CDK6 controls the cell cycle, and dysregulation of CDK6 is associated with bladder cancer progression [19]. To further validate the association between the GAS5 and CDK6, we next performed a RIP assay with an antibody against CDK6 on RT4 cellular extracts. Consistently, we observed a significantly higher enrichment level of GAS5 with the CDK6 antibody compared with the non-specific IgG control antibody (Figure 3B). Knockdown of GAS5 significantly increases CDK6 mRNA and protein levels in bladder cancer cell lines (Figure 3C and D), but downregulation of GAS5 don’t change CDK2 and CDK4 expression level (data not shown). Furthermore, overexpression of GAS5 remarkably inhibits CDK6 expression in bladder cancer cell lines (Figure 3E). *In vivo*, a significant negative correlation is also observed between the GAS5 levels and the CDK6 levels in cancer tissues (*r* ^2^ = 0.168, *p* = 0.013, Figure 3F). We further investigate the role of GAS5 in the regulation of cell apoptosis and cell cycle. Figure 4A showed that GAS5-siRNA treatment inhibits cell apoptotic. Then the GAS5 or CDK6 is knocked down and cell cycle is analyzed by flow cytometry. Compared with control siRNA, GAS5 downregulation displays a decreased percentage of cells in G0/G1 phase and more cells in S phase (Figure 4B). The quantitative analysis also reveals a significant decrease in the cell population in G0/G1 phase in cells tranfected with GAS5-siRNA (Figure 4B). More important, knockdown of CDK6 by specific siRNAs reduces the percentage of cells in S phase in GAS5-siRNA-treated cells (Figure S1, Figure 4B). These data indicate that GAS5 regulates bladder cancer cell cycle, at least in part, by the regulation of CDK6.

**Table 2:** Mass spectrometry analysis of the proteins pull-down by GAS5.

Hits	Protein Mass (Da)	Gene name	Relative abundance(%)
1	36425.1	malate dehydrogenase	30.4
2	36937.4	cyclin-dependent kinase 6	27.2
3	38417.8	Glycerol-3-phosphate dehydrogenase 1-like	5.3
4	42255.9	glucocorticoid receptor	17.1
5	28352.3	peroxisomal biogenesis factor 11A	3.7
6	49882.9	ZNF154 protein	2.4
7	41756.7	histone deacetylase 8 isoform 1	2.8
8	43537.8	putative glycolipid transfer protein	4.0
9	33894.2	histone H1 transcription factor large subunit 2A	6.5
10	37273.2	ephrin type-A receptor 6 isoform b	0.6

### GAS5 Decreases Bladder Cancer Cell Proliferation by Regulating CDK6

**Figure 5:**
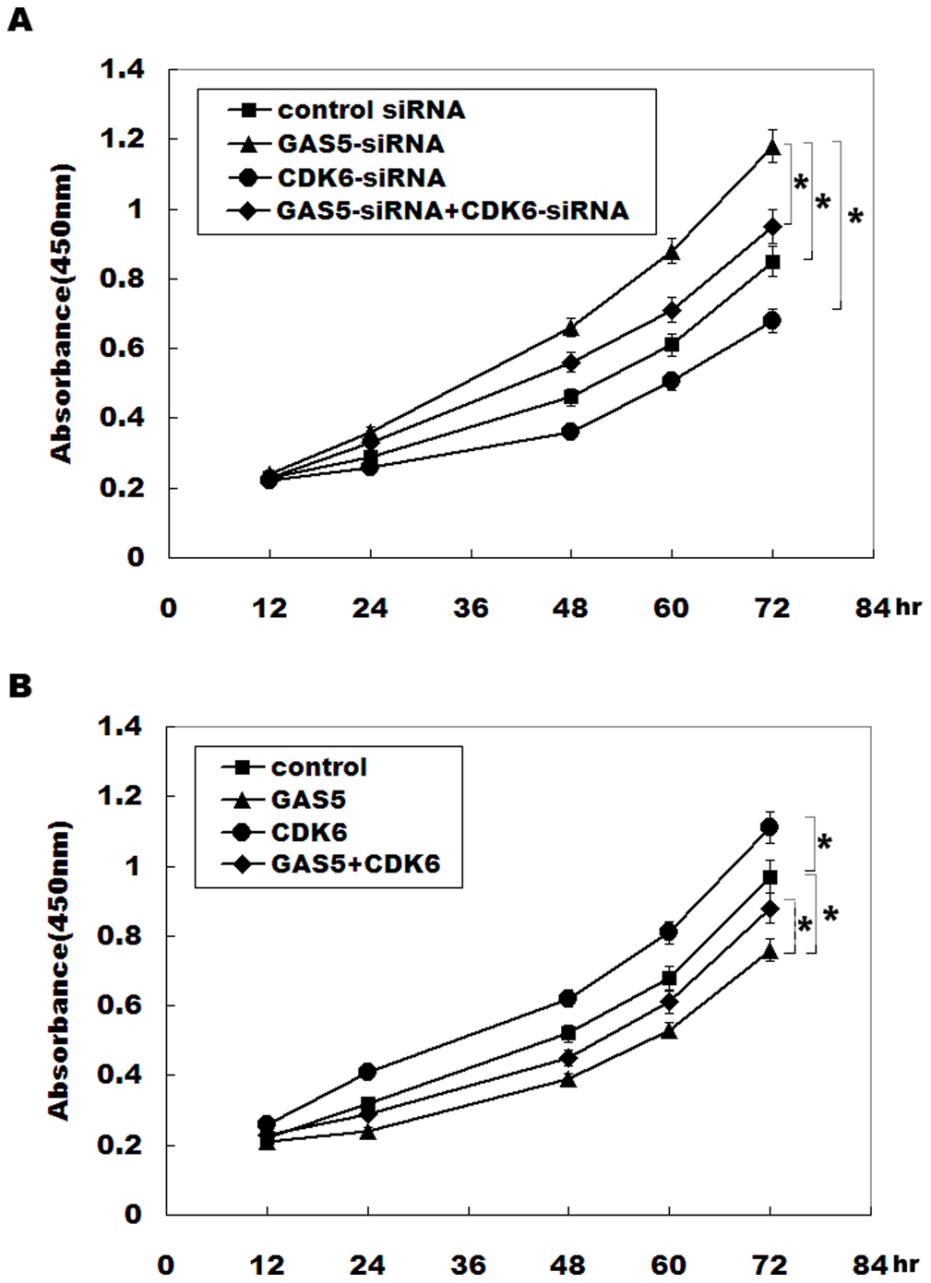
GAS5 inhibits bladder cancer cell proliferation, partly by regulating CDK6. (A) RT4 cells were treated with GAS5-siRNA and CDK6-siRNA, and the numbers of cells per well were measured by the absorbance (450 nm) of reduced WST-8. *p<*0.05*. (B) RT4 cells were overexpressed with GAS5 and CDK6, and the numbers of cells per well were measured by the absorbance (450 nm) of reduced WST-8. *p<*0.05*.

Downregulated GAS5 increases bladder cancer cell proliferation, and a significant negative correlation is observed between the GAS5 and the CDK6. We therefore thought that the role of GAS5 in regulating bladder cancer cell proliferation is mediated by modulating CDK6 expression. Consistent with previous studies, knockdown of CDK6 inhibits RT4 cell proliferation (Figure 5A). Furthermore, RT4 cell proliferation is partially suppressed by CDK6 knockdown in GAS5-siRNA-treated cells (Figure 5A). In GAS5-overexpressing cells, forced expression of CDK6 results in a restored cell proliferation (Figure 5B). These data confirm that GAS5 decreases bladder cancer progression, at least in part, by regulating CDK6 expression.

## Discussion

Recent studies show that the human transcriptome is more complex than a collection of protein-coding genes; showing extensive antisense and non-coding RNA (ncRNA) expression [20], [21], [22]. Although initially argued to be spurious transcriptional noise, new evidence demonstrates that ncRNAs (such as microRNAs and lncRNAs) participate in the regulation of cellular development, cell growth and human diseases [23], [24]. Recent studies are beginning to unravel their importance in tumorigenesis. For example, lncRNA-H19 level is remarkably elevated in a large number of human cancers [25], [26], and H19 overexpression confers a growth advantage on cancer cells [27].

GAS5 is a newly identified lncRNA involved in the regulation of cell cycle [16], [28]. Mourtada-M *et al* showed that GAS5 plays a crucial role in normal growth arrest in both T-cell lines and non-transformed lymphocytes [16]. GAS5 overexpression results in both an increase in apoptosis and a reduction in the rate of progression through the cell-cycle, whereas GAS5 knockdown inhibits apoptosis and maintains a more rapid cell cycle, indicating that GAS5 expression is necessary to normal growth arrest in T-cell lines and human peripheral blood T-cells [16]. GAS5 levels are also significantly reduced in breast cancer samples [17]. Based on these findings, we speculated whether expression of the GAS5 is abnormal and dysregulated GAS5 regulates cell proliferation in bladder cancer. In the present study, we identify that the GAS5 expression is commonly downregulated in most bladder cancer specimens and in bladder cancer cell lines. GAS5 inhibition contributes to bladder cancer cell proliferation, whereas overexpression of GAS5 inhibits cell proliferation. Previous studies showed that GAS5 binds to the DNA-binding domain of the glucocorticoid receptor (GR) by acting as a decoy glucocorticoid response element (GRE), thus competing with DNA GREs for binding to the GR. Thus GAS5 is a “riborepressor” of the GR, influencing cell survival and metabolic activities during starvation by modulating the transcriptional activity of the GR [29]. Here we performed a RNA pull-down assay to investigate the potential mechanism of GAS5 in regulating cell growth. Our data revealed that GAS5 could combine with the cyclin-dependent kinase 6 (CDK6), and further repress the expression of CDK6. Therefore, downregulation of GAS5 increases CDK6 expression in bladder cancer cells. GAS5 inhibition induces a significant decrease in G0/G1 phase and an increase in S phase by CDK6-dependent manner. Gain-of-function and loss-of-function studies confirmed that GAS5 regulates cell cycle and inhibits bladder cancer cell proliferation, partly by regulating CDK6 expression.

Tumor development and progression have been shown to be dependent on cellular accumulation of various epigenetic and genetic events, including alterations in the cell-cycle machinery at G1/S checkpoint [30]. The G1/S phase transition is primarily regulated by D-type cyclins (D1, D2, or D3) in complex with CDK4/CDK6, and E-type cyclins (E1, or E2) in complex with CDK2 [30]. CDK6 is showed to be overexpressed in bladder cancer [31], and the concept has emerged that Rb phosphorylation by CDK4/6 leads not only to critical E2F-dependent transcription of essential cell cycle enzymes and regulators but also to assembly of the pre-replication complex in G1 phase [31]. Therefore, GAS5/CDK6 pathway may play an important role in regulating cancer development and progression.

### Conclusions

These results suggest that downregulation of GAS5 increases bladder cancer cell proliferation, at least in part, by regulating CDK6. Our findings contributes to a better understanding of the importance of the dysregulated lncRNAs in bladder cancer progression and provides a rationale for the potential development of lncRNA-based targeted approaches for the treatment of bladder cancer.

## Supporting Information

Figure S1 **Western blot analysis of CDK6 protein level was performed in bladder cancer cells treated with CDK6-siRNA.**
(TIF)

## References

[1] Bo J, Yang G, Huo K, Jiang H, Zhang L, et al (2011) microRNA-203 suppresses bladder cancer development by repressing bcl-w expression. FEBS J 278: 786–792. 21205209 10.1111/j.1742-4658.2010.07997.x

[2] Lacey JV Jr, Devesa SS, Brinton LA (2002) Recent trends in breast cancer incidence and mortality. Environ Mol Mutagen 39: 82–88. 11921173 10.1002/em.10062

[3] Seiler R, von Gunten M, Thalmann GN, Fleischmann A (2010) Pelvic lymph nodes: distribution and nodal tumour burden of urothelial bladder cancer. J Clin Pathol 63: 504–507. 20364028 10.1136/jcp.2009.075077 PMC2981017

[4] Hirata H, Ueno K, Shahryari V, Tanaka Y, Tabatabai ZL, et al (2012) Oncogenic miRNA-182–5p targets Smad4 and RECK in human bladder cancer. PLoS One 7: e51056. 23226455 10.1371/journal.pone.0051056 PMC3511415

[5] Pasin E, Josephson DY, Mitra AP, Cote RJ, Stein JP (2008) Superficial bladder cancer: an update on etiology, molecular development, classification, and natural history. Rev Urol 10: 31–43. 18470273 PMC2312342

[6] Said N, Sanchez-Carbayo M, Smith SC, Theodorescu D (2012) RhoGDI2 suppresses lung metastasis in mice by reducing tumor versican expression and macrophage infiltration. J Clin Invest 122: 1503–1518. 22406535 10.1172/JCI61392 PMC3314474

[7] Zhang X, Gejman R, Mahta A, Zhong Y, Rice KA, et al (2010) Maternally expressed gene 3, an imprinted noncoding RNA gene, is associated with meningioma pathogenesis and progression. Cancer Res 70: 2350–2358. 20179190 10.1158/0008-5472.CAN-09-3885 PMC2987571

[8] Gutschner T, Diederichs S (2012) The hallmarks of cancer: a long non-coding RNA point of view. RNA Biol 9: 703–719. 22664915 10.4161/rna.20481 PMC3495743

[9] Luo M, Li Z, Wang W, Zeng Y, Liu Z, et al (2013) Upregulated H19 contributes to bladder cancer cell proliferation by regulating ID2 expression. FEBS J 280: 1709–1716. 23399020 10.1111/febs.12185

[10] Luo M, Li Z, Wang W, Zeng Y, Liu Z, et al (2013) Long non-coding RNA H19 increases bladder cancer metastasis by associating with EZH2 and inhibiting E-cadherin expression. Cancer Lett 333: 213–221. 23354591 10.1016/j.canlet.2013.01.033

[11] Woo CJ, Kingston RE (2007) HOTAIR lifts noncoding RNAs to new levels. Cell 129: 1257–1259. 17604716 10.1016/j.cell.2007.06.014

[12] Gupta RA, Shah N, Wang KC, Kim J, Horlings HM, et al (2010) Long non-coding RNA HOTAIR reprograms chromatin state to promote cancer metastasis. Nature 464: 1071–1076. 20393566 10.1038/nature08975 PMC3049919

[13] Kogo R, Shimamura T, Mimori K, Kawahara K, Imoto S, et al (2011) Long noncoding RNA HOTAIR regulates polycomb-dependent chromatin modification and is associated with poor prognosis in colorectal cancers. Cancer Res 71: 6320–6326. 21862635 10.1158/0008-5472.CAN-11-1021

[14] Tano K, Akimitsu N (2012) Long non-coding RNAs in cancer progression. Front Genet 3: 219. 23109937 10.3389/fgene.2012.00219 PMC3479403

[15] Zhou Y, Zhang X, Klibanski A (2012) MEG3 noncoding RNA: a tumor suppressor. J Mol Endocrinol 48: R45–53. 22393162 10.1530/JME-12-0008 PMC3738193

[16] Mourtada-Maarabouni M, Hedge VL, Kirkham L, Farzaneh F, Williams GT (2008) Growth arrest in human T-cells is controlled by the non-coding RNA growth-arrest-specific transcript 5 (GAS5). J Cell Sci 121: 939–946. 18354083 10.1242/jcs.024646

[17] Mourtada-Maarabouni M, Pickard MR, Hedge VL, Farzaneh F, Williams GT (2009) GAS5, a non-protein-coding RNA, controls apoptosis and is downregulated in breast cancer. Oncogene 28: 195–208. 18836484 10.1038/onc.2008.373

[18] Kapinas K, Kessler CB, Ricks T, Gronowicz G, Delany AM (2010) miR-29 modulates WNT signaling in human osteoblasts through a positive feedback loop. J Biol Chem 285: 25221–25231. 20551325 10.1074/jbc.M110.116137 PMC2919085

[19] Lodygin D, Tarasov V, Epanchintsev A, Berking C, Knyazeva T, et al (2008) Inactivation of miR-34a by aberrant CpG methylation in multiple types of cancer. Cell Cycle 7: 2591–2600. 18719384 10.4161/cc.7.16.6533

[20] Kapranov P, Willingham AT, Gingeras TR (2007) Genome-wide transcription and the implications for genomic organization. Nat Rev Genet 8: 413–423. 17486121 10.1038/nrg2083

[21] Washietl S, Hofacker IL, Lukasser M, Huttenhofer A, Stadler PF (2005) Mapping of conserved RNA secondary structures predicts thousands of functional noncoding RNAs in the human genome. Nat Biotechnol 23: 1383–1390. 16273071 10.1038/nbt1144

[22] Gibb EA, Brown CJ, Lam WL (2011) The functional role of long non-coding RNA in human carcinomas. Mol Cancer 10: 38. 21489289 10.1186/1476-4598-10-38 PMC3098824

[23] Mercer TR, Dinger ME, Mattick JS (2009) Long non-coding RNAs: insights into functions. Nat Rev Genet 10: 155–159. 19188922 10.1038/nrg2521

[24] Wilusz JE, Sunwoo H, Spector DL (2009) Long noncoding RNAs: functional surprises from the RNA world. Genes Dev 23: 1494–1504. 19571179 10.1101/gad.1800909 PMC3152381

[25] Fellig Y, Ariel I, Ohana P, Schachter P, Sinelnikov I, et al (2005) H19 expression in hepatic metastases from a range of human carcinomas. J Clin Pathol 58: 1064–1068. 16189152 10.1136/jcp.2004.023648 PMC1770739

[26] Matouk IJ, Mezan S, Mizrahi A, Ohana P, Abu-Lail R, et al (2010) The oncofetal H19 RNA connection: hypoxia, p53 and cancer. Biochim Biophys Acta 1803: 443–451. 20117150 10.1016/j.bbamcr.2010.01.010

[27] Berteaux N, Lottin S, Monte D, Pinte S, Quatannens B, et al (2005) H19 mRNA-like noncoding RNA promotes breast cancer cell proliferation through positive control by E2F1. J Biol Chem 280: 29625–29636. 15985428 10.1074/jbc.M504033200

[28] Coccia EM, Cicala C, Charlesworth A, Ciccarelli C, Rossi GB, et al (1992) Regulation and expression of a growth arrest-specific gene (gas5) during growth, differentiation, and development. Mol Cell Biol 12: 3514–3521. 1630459 10.1128/mcb.12.8.3514-3521.1992 PMC364604

[29] Kino T, Hurt DE, Ichijo T, Nader N, Chrousos GP (2010) Noncoding RNA gas5 is a growth arrest- and starvation-associated repressor of the glucocorticoid receptor. Sci Signal 3: ra8. 20124551 10.1126/scisignal.2000568 PMC2819218

[30] Sun F, Fu H, Liu Q, Tie Y, Zhu J, et al (2008) Downregulation of CCND1 and CDK6 by miR-34a induces cell cycle arrest. FEBS Lett 582: 1564–1568. 18406353 10.1016/j.febslet.2008.03.057

[31] Wang G, Zheng L, Yu Z, Liao G, Lu L, et al (2012) Increased cyclin-dependent kinase 6 expression in bladder cancer. Oncol Lett 4: 43–46. 22807957 10.3892/ol.2012.695 PMC3398375

[32] Taganov KD, Boldin MP, Chang KJ, Baltimore D (2006) NF-kappaB-dependent induction of microRNA miR-146, an inhibitor targeted to signaling proteins of innate immune responses. Proc Natl Acad Sci U S A 103: 12481–12486. 16885212 10.1073/pnas.0605298103 PMC1567904

