# Supplementary figures and images for: Downregulation of GAS5 Promotes Bladder Cancer Cell Proliferation, Partly by Regulating CDK6
Source: PLoS One. 2013 Sep 17;8(9):e73991. doi: 10.1371/journal.pone.0073991 (PMC3775789; doi:10.1371/journal.pone.0073991)

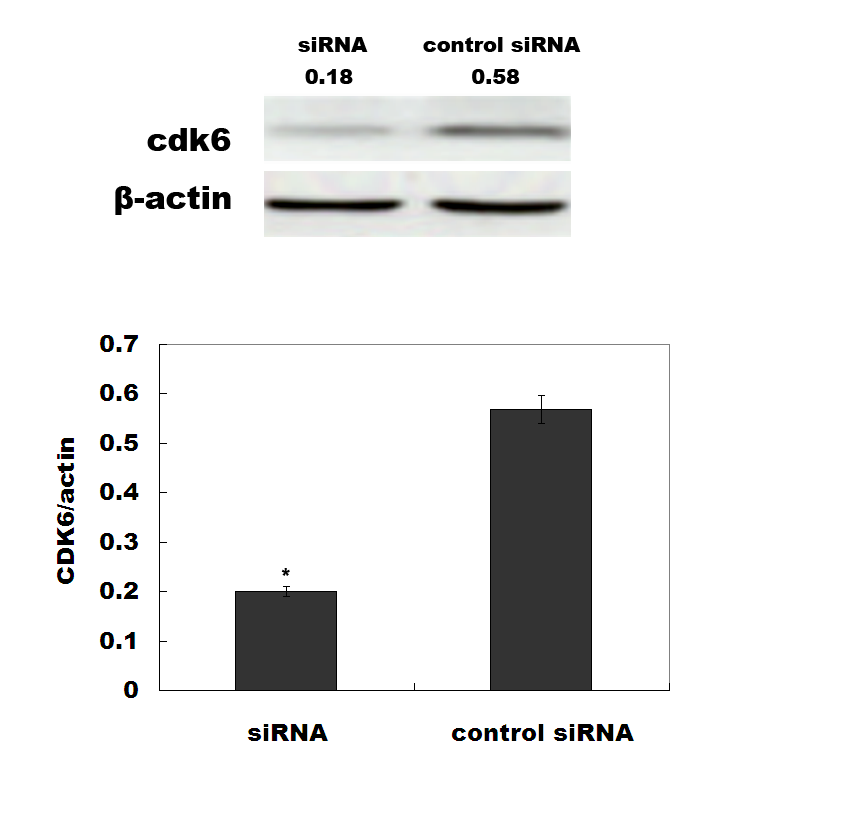

Supplement: Figure S1 — Western blot analysis of CDK6 protein level was performed in bladder cancer cells treated with CDK6-siRNA. (TIF) [file pone.0073991.s001.tif]
